# Validity and Reproducibility of a Spanish Dietary History

**DOI:** 10.1371/journal.pone.0086074

**Published:** 2014-01-20

**Authors:** Pilar Guallar-Castillón, Jon Sagardui-Villamor, Teresa Balboa-Castillo, Aleix Sala-Vila, Mª José Ariza Astolfi, Mª Dolores Sarrión Pelous, Luz María León-Muñoz, Auxiliadora Graciani, Martín Laclaustra, Cristina Benito, José Ramón Banegas, Fernando Rodríguez Artalejo

**Affiliations:** 1 Department of Preventive Medicine and Public Health, School of Medicine, Universidad Autónoma de Madrid/IdiPAZ, Madrid, Spain; 2 CIBER de Epidemiología y Salud Pública (CIBERESP), Instituto de Salud Carlos III (ISCIII), Madrid, Spain; 3 Centro de Salud de Villanueva del Pardillo, Madrid, Spain; 4 Department of Public Health, School of Medicine, Universidad de la Frontera, Temuco, Chile; 5 Lipid Clinic, Endocrinology and Nutrition Service, Biomedical Research Institute August Pi i Sunyer (IDIBAPS), Hospital Clínic, Barcelona, Spain; 6 CIBER de Fisiopatología de la Obesidad y Nutrición (CIBERobn), Instituto de Salud Carlos III (ISCIII), Madrid, Spain; 7 Hospital Universitario La Paz, Department of Biochemistry, Madrid, Spain; 8 Centro Nacional de Investigaciones Cardiológicas (CNIC), Madrid, Spain; Paris Institute of Technology for Life, Food and Environmental Sciences, France

## Abstract

**Objective:**

To assess the validity and reproducibility of food and nutrient intake estimated with the electronic diet history of ENRICA (DH-E), which collects information on numerous aspects of the Spanish diet.

**Methods:**

The validity of food and nutrient intake was estimated using Pearson correlation coefficients between the DH-E and the mean of seven 24-hour recalls collected every 2 months over the previous year. The reproducibility was estimated using intraclass correlation coefficients between two DH-E made one year apart.

**Results:**

The correlations coefficients between the DH-E and the mean of seven 24-hour recalls for the main food groups were cereals (r = 0.66), meat (r = 0.66), fish (r = 0.42), vegetables (r = 0.62) and fruits (r = 0.44). The mean correlation coefficient for all 15 food groups considered was 0.53. The correlations for macronutrients were: energy (r = 0.76), proteins (r = 0.58), lipids (r = 0.73), saturated fat (r = 0.73), monounsaturated fat (r = 0.59), polyunsaturated fat (r = 0.57), and carbohydrates (r = 0.66). The mean correlation coefficient for all 41 nutrients studied was 0.55. The intraclass correlation coefficient between the two DH-E was greater than 0.40 for most foods and nutrients.

**Conclusions:**

The DH-E shows good validity and reproducibility for estimating usual intake of foods and nutrients.

## Introduction

Diet has been associated with the risk of numerous chronic diseases. However, measuring the diet is difficult, especially the usual diet, which is the most relevant. The main difficulty is remembering the type and amount of food consumed; added to this is the inter- and intra-individual variability in food intake, which is accentuated by variation in seasonal intake [1].

Epidemiological studies have mainly used two instruments to collect the usual diet. The most commonly used has been the semiquantitative food frequency questionnaire (FFQ) [2], [3]. It consists of a list of foods in which the respondent must choose the frequency of consumption of each, in predefined categories. Its principal advantage is that it can be self-administered and is easy to use. The second instrument is the diet history (DH), which consists of a structured interview following each intake occasion, from breakfast to bedtime [4]–[7]. Its main advantage is that it reports the distribution of food consumption throughout the day, the way it is cooked, seasonal consumption, and the variations in consumption on weekdays and weekends. The most important limitation is that it must be performed by a trained interviewer and is time-consuming. At times the DH has been used as the reference method to validate other instruments for collecting the usual diet [8], [9].

Fifteen years ago the EPIC group in Spain designed and validated a DH (DH-EPIC) [10]–[12]. The DH-EPIC has made it possible, for example, to study different cooking methods and their association with obesity [13] and the incidence of coronary disease [14] in Spain. In the context of the Study on Nutrition and Cardiovascular Risk in Spain (ENRICA) [15] we recently developed a new electronic DH based on the DH-EPIC. This new instrument, DH-ENRICA® (DH-E), includes a larger number of foods and nutrients than the DH-EPIC, incorporates new photographs to estimate portion sizes, includes new traditional dishes and cooking methods characteristic of Spanish cuisine, and takes account of the extent to which foods are processed. It also includes a dictionary of synonyms for foods from the different regions of Spain.

The aim of this study was to examine the validity and reproducibility of food and nutrient intake estimated with the DH-E.

## Methods

### Ethics Statement

The study was approved by the Ethics Committee of the Hospital Universitario “La Paz”. Written informed consent was obtained from all study participants.

### Study Design

**Figure 1:**
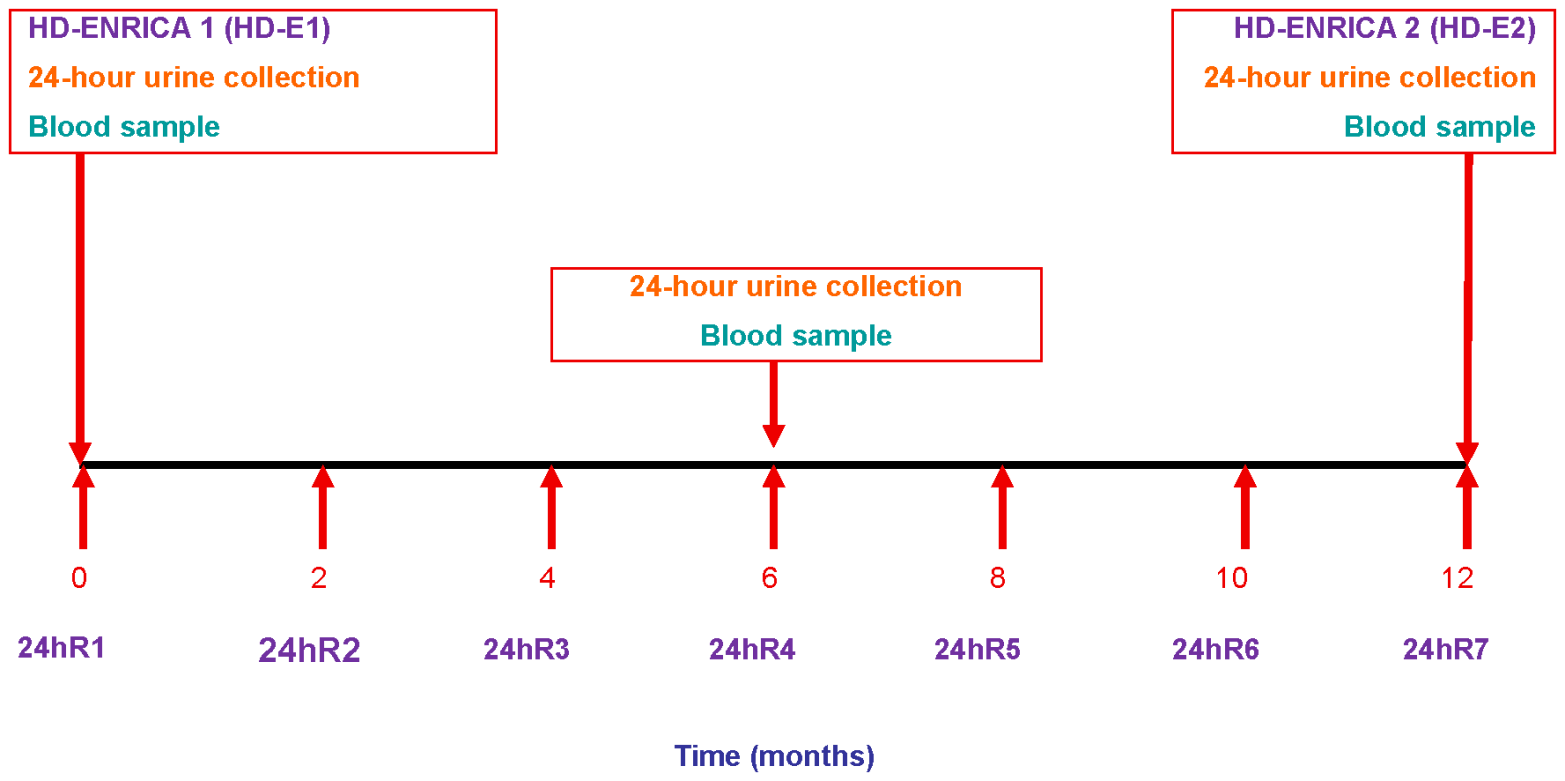
Design of validation study of diet history-ENRICA (DH-E). Footnote: 24hR: 24-hour recall.

We assessed the validity of the DH-E compared to the mean of seven 24-hour recall periods (the gold standard) collected every 2 months during one year (Figure 1). Individuals were considered to have completed the study when they had provided the DH-E at month 0 and month 12 and, additionally, seven 24-hour recalls during the year. All participants completed at least one 24-hour recall on a weekend. The DH-E was also validated against dietary biomarkers. For this purpose, samples of blood and 24-hour urine were collected at baseline, at 6 months and at 12 months.

We assessed the reproducibility between the DH-E made at month 0 and at month 12. Individuals who reported having changed their diet during the year were excluded from the analysis.

### Study Participants

The participants were 132 persons aged 18 and over recruited by the physicians of the Primary Care Center of Villanueva del Pardillo (Madrid). The exclusion criteria were taking vitamin supplements, having diabetes, and planning a change of diet within the next year. Of the 132 persons who agreed to participate, 104 provided complete information at 12 months (losses of 23.5%). Of these, 3 persons reported having changed their diet during the study year, consequently the analyses were made with 101 individuals. The study took place from April 2010 to December 2011.

### The DH-E Interview

The DH-E is a computerized questionnaire administered by a trained interviewer. The interview has two parts. First, the subject is requested to indicate all the foods usually consumed in the previous year. The interview begins with the question: “What do you usually have to eat when you get up?” and continues asking about usual consumption on the six main intake occasions (when getting up, breakfast, mid-morning, lunch, mid-afternoon and dinner) and between those occasions, like snacking, before bedtime and going out for a drink. To facilitate reporting of food consumed at lunch and dinner, we asked about the first and second course, dessert, beverage consumption, bread, etc.

In the interview, respondents are asked about food consumption during the week and on the weekend, as well as seasonal variations. All the information refers to a typical week, for which conversion factors are used that consider the weekly frequency of consumption of a food and the number of months in which it is consumed during the year. A food was considered to be “usually consumed” when it was eaten at least once every 15 days.

The second part of the interview asks about the food groups that were not reported, and about specific foods that are difficult to report spontaneously, like alcoholic beverages or bread. It begins with questions like “Do you like to eat bread with your meals?” This helps to clarify or verify the information on some foods collected in the first part of the interview.

### The DH-E Instrument

The DH-E collects standardized information on 861 foods that can be cooked in 29 different ways (including mixed forms of cooking and food preservation methods). The software includes aids for the correct classification of some foods (e.g., fermented milk or butter and margarine). It also includes 127 sets of digitized photographs to estimate the size of food portions; specifically, for each individual food or food mixture the respondent is presented with photos of three portion sizes (small, large and medium), which allows classification in 7 different sizes. When no photo of a food was available, the amount consumed was estimated with natural units or household measures; the DH-E includes 122 household measures (e.g., a carton of yogurt = 125 g). The amount of oil added to salads or vegetables was evaluated by the respondent’s estimation of the number of spoonfuls of oil added, or of how oily the foods were.

The DH-E includes 184 recipes for dishes commonly eaten in Spain or typical of each region. The recipes are converted into simple foods based on the proportion and combination reported by the respondent or according to standard compositions.

The DH-E collects information on the degree to which foods are processed, calculates the annual frequency of consumption based on seasonal consumption, and applies fat absorption coefficients for foods that are fried, coated, breaded or sautéed. Furthermore, it automatically converts the foods to nutrients using food composition tables from Spain [16]–[21] and other countries [22]–[26]. The DH-E also asks about foods consumed in association with other foods, but that are not cooked together (e.g., a person who reports drinking coffee is asked about consumption of sugar or other sweeteners).

Finally, to facilitate quality control of the diet interview, the DH-E generates alerts when unacceptable values are registered for energy intake, or when foods that are generally part of the main eating occasions are not reported.

### Interviewer Training

A single interviewer received a standardized training and conducted all the study interviews. The training covered three stages. The first lasted 2 days and included theoretical explanations and practical exercises on the diet interview and software management. Evaluation of the interviewer consisted of conducting a simulated diet interview, which required recording of the variation in food consumption, both seasonal and between weekdays and weekends. In the second stage, the interviewer conducted 8–10 interviews with volunteers who reported their actual diet. Finally, the interviewer conducted a real, unprepared interview under the supervision of the trainer. After this final interview, the interviewer was certified.

### Reference Method

The reference method was the mean of seven 24-hour recall periods conducted in all seasons of the year, and on both weekdays and weekends. Each participant also had to complete a questionnaire on the amount of food, how it was cooked and the time of consumption. Participants were told not to modify their usual way of eating because of the recall.

### Processing of Biological Samples and Determination of Dietary Biomarkers

Subjects were instructed on how to collect a 24-hour urine sample, beginning early in the morning (after emptying the bladder to eliminate urine formed during the night) and ending the following morning at the same time (including the urine formed during the night). Participants kept the urine refrigerated at 4°C and took it to the Primary Care Center where they were asked about possible problems in the completeness and storage of urine.

In the Primary Care Center, both 12-h fasting serum and 0.1% EDTA blood were collected and processed while protected from the light. They were frozen at −80° in various aliquots immediately after extraction. Whole blood cell membrane fatty acids were determined by gas chromatography (Agilent 6890 Gas Chromatograph HP 6890 with capillary column, autosampler and flame ionization detector) as described [27]. Vitamins A and E were measured by high-resolution liquid chromatography (HPLC-Agilent Technologies Series 1200), and vitamin C by spectrofluorimetry (Perkin Elmer model LS3). Total cholesterol was measured by an enzymatic method using cholesterol-esterase and cholesterol-oxidase. Vitamin E was divided by total cholesterol to take their correlation into account. Serum calcium was determined by the Arsenazo III method. Ions were measured by indirect potentiometry and urea nitrogen by urease.

### Other Variables

In addition to sociodemographic variables and smoking status, weight and height were measured under standardized conditions using electronic scales and wall-mounted stadiometers [28]. Subjects were classified into three groups: normal weight (BMI <25.0), overweight (BMI 25.0–29.9) and obese (BMI ≥30). Waist and hip circumference were measured with a flexible, non-elastic tape [28]. Physical activity was assessed with a validated questionnaire that includes both leisure-time activity and housework [29].

### Statistical Analysis

The validity of food and nutrient intake obtained with DH-E was estimated using Pearson correlation coefficients between the DH-E conducted at the end of the study (DH-E2) and the mean of the seven 24-hour recalls conducted throughout the study. Food and nutrient intake was log transformed to improve the normality of the distribution. Adjusted correlation coefficients were also calculated for total energy intake using the residuals method [30]. To correct intra-individual error in the measurement of the seven 24-hour recalls, the correlation coefficients were multiplied by a de-attenuation factor (1+(σ_w_^2^/σ_b_^2^)/n)^0.5^, where σ_w_^2^ is the intra-individual variance, σ_b_^2^ is the inter-individual variance, and n is the number of repeated measures (in this case seven). The intra- and inter-individual components of the variance were calculated under a random effects model [31].

Another way of assessing the validity of the DH-E is by the classification error of subjects according to the 24-hour recall values. For this purpose we calculated the quintiles of the distribution of food and nutrient intake. Gross misclassification was considered to occur when a subject in the lowest DH-E quintile was in the highest quintile of the 24-hour recalls and vice versa. In contrast, classification was considered to be correct when a subject was in the same or adjacent quintiles of the distribution of DH-E and the 24-hour recalls.

The estimated nutrient intake with DH-E was correlated with the mean of the last three measures of dietary biomarkers (measured at 0, 6 and 12 months) using Pearson correlation coefficients.

The reproducibility of the DH-E was estimated with Pearson correlation coefficients and with the intra-class correlation coefficient (ICC) between the DH-E at baseline (DH-E1) and at the end of the study (DH-E2), assuming a fixed effects model.

The analyses were performed with SAS version 9.1.

## Results

The mean age of study participants was 44 years, and 74% were women. Most had secondary or higher education, were non-smokers and had a normal BMI (Table 1).

**Table 1:** Baseline characteristics of study participants (N = 101).

	Mean ± SD*or percentage
**Age,** mean ± SD*	43.6±13.6
**Sex**, %	
Men	25.5
**Educational level**, %	
No education	7.8
Primary	10.8
Secondary and tertiary	81.4
**Smoking**, %	
Never smoker	44.4
Former smoker	29.3
Current smoker	26.3
**Weight status**, %	
Normal (BMI <25 Kg/)	41.2
Overweight (BMI 25.0–29.9 Kg/m^2^)	32.4
Obese (BMI ≥30 Kg/m^2^)	26.4
**Waist circumference,** mean ± SD*	84.9±12.5
**Hip circumference,** mean ± SD*	102.9±13.0
**Physical activity (METs),** mean ± SD*	66.7±46.9
Leisure time	37.1±37.3
Housework	29.6±33.1

SD: Standard deviation.

No important differences were observed in the absolute measures for food and nutrients according to the information collection method (Table 2). The most notable differences between the mean of seven 24-hour recalls and the DH-E were found for intake of vegetables and docosapentanoic acid (DPA, 22∶5*n*-3), for which consumption was overestimated. However, consumption of retinoids was underestimated.

**Table 2:** Food and nutrient intake according to DH-E1 (at study baseline), mean of seven 24-hour recalls during one year, and DH-E2 (at 12 months from baseline). (N = 101).

	Diet history 1(DH-E1)		Mean of seven24-h recalls(7 24 hR)		Diet history 2(DH-E2)	
	Mean	SD	Mean	SD	Mean	SD
**Food groups**						
Cereals (g/d)	276.5	105.3	252.9	82.7	259.3	80.8
Milk (g/d)	339.6	175.2	321.6	155.7	358.6	168.9
Meat (g/d)	135.0	70.3	142.4	68.3	134.0	53.2
Eggs (g/d)	22.2	14.8	27.1	20.8	23.0	22.2
Fish (g/d)	62.4	43.2	52.1	37.5	55.7	33.0
Oils and fats (g/d)	33.4	11.4	37.2	13.4	37.7	11.9
Vegetables (g/d)	233.7	112.0	187.3	105.9	233.6	94.2
Legumes (g/d)	45.9	59.1	44.1	48.1	42.8	55.3
Tubers (g/d)	57.3	49.5	50.3	29.4	47.8	25.8
Fruits (g/d)	268.7	170.5	246.3	131.6	253.6	134.5
Dried fruits and nuts (g/d)	10.8	16.6	5.6	10.4	6.0	9.2
Chocolate and similar (g/d)	10.5	16.0	7.5	11.4	8.9	12.9
Coffee, cocoa and infusions (ml/d)	196.8	173.9	185.2	145.9	196.8	189.7
Soft drinks (ml/d)	163.6	278.1	158.5	221.3	138.0	216.7
Alcoholic beverages (ml/d)	97.2	138.9	81.4	111.3	81.6	88.4
**Nutrients**						
Energy (kcal/d)	2402.6	539.6	2248.1	386.8	2279.7	400.0
Total protein (g/d)	115.0	18.4	92.7	18.3	93.7	16.6
Animal protein (g/d)	65.6	20.9	64.9	18.0	64.3	15.1
Vegetable protein (g/d)	32.5	9.9	27.9	5.9	29.3	6.2
Fats (g/d)	106.7	29.6	106.0	24.4	105.8	25.4
Saturated fatty acids (g/d)	32.4	11.3	32.2	9.2	32.0	9.8
Monounsaturated fatty acids (g/d)	45.8	11.8	46.9	10.3	47.1	10.0
Polyunsaturated fatty acids (g/d)	20.6	9.7	18.1	7.4	18.3	7.7
Linoleic acid (18∶2*n*-6) (g/d)	18.0	9.3	15.8	7.1	16.1	7.3
α-linolenic acid (18∶3*n*-3) (g/d)	1.3	0.5	1.2	0.4	1.3	0.5
Eicosapentanoic acid (20∶5*n*-3) (g/d)	0.21	0.21	0.18	0.18	0.18	0.15
Docosapentanoic acid (22∶5*n*-3), (g/d)	0.14	0.10	0.11	0.08	0.14	0.08
Docosahexanoic acid (22∶6*n*-3), (g/d)	0.37	0.34	0.29	0.29	0.32	0.23
*Trans* fatty acids (g/d)	2.8	2.2	2.7	1.5	2.5	1.2
Cholesterol (mg)	365.6	130.7	371.0	114.7	375.7	114.3
Total carbohydrates (g/d)	252.2	72.5	220.4	43.4	227.6	46.1
Sugars (g/d)	118.5	44.2	103.1	23.6	104.4	29.3
Polysaccharides (g/d)	132.5	47.7	117.3	30.5	123.3	34.4
Ethanol (g/d)	6.5	9.5	5.6	7.6	5.6	6.4
Fiber (g/d)	23.4	7.4	20.0	5.2	21.8	5.0
Caffeine (mg/d)	142.4	147.4	120.4	86.0	117.9	195.4
Sodium (mg/d)	2940.8	951.2	2848.3	757.6	2784.7	714.8
Potassium (mg/d)	3421.6	736.8	3107.4	508.6	3192.0	525.4
Calcium (mg/d)	943.9	356.4	860.5	202.5	922.4	240.2
Magnesium (mg/d)	346.8	100.1	303.6	58.8	312.6	55.4
Phosphorus (mg/d)	1484.3	351.4	1403.8	251.6	1419.8	244.3
Iron (mg/d)	27.2	1.4	12.6	2.6	12.2	2.2
Zinc (mg/d)	9.5	2.2	9.0	1.7	9.0	1.7
Selenium (µg/d)	136.4	42.2	126.4	42.4	123.0	28.3
Iodine (µg/d)	177.5	118.4	175.7	88.7	167.0	113.3
Vitamin A (µg/d)	900.7	422.1	947.1	625.5	903.8	265.0
Retinoids (µg/d)	380.2	276.3	457.5	573.0	355.9	172.8
Carotenoids (µg/d)	3179.1	1682	2937.6	1721.0	3288.1	1401.0
Vitamin D (µg/d)	19.4	1.6	3.0	1.7	2.9	1.3
Vitamin E (mg/d)	14.4	7.9	12.6	5.6	12.7	5.1
Thiamine (mg/d)	1.6	0.8	1.5	0.4	1.4	0.4
Riboflavin (mg/d)	1.8	0.6	1.8	0.4	1.7	0.4
Niacin (mg/d)	23.4	6.9	22.5	5.5	21.8	4.5
Vitamin B6 (mg/d)	2.4	2.0	2.0	0.4	2.0	0.4
Folic acid (µg/d)	351.0	105.0	312.9	86.2	322.1	80.5
Vitamin B12 (µg/d)	8.1	2.2	6.5	4.2	5.5	2.1
Vitamin C (mg/d)	141.3	68.9	130.3	57.5	131.7	55.3

SD: Standard deviation.

### Validity of Food and Nutrient Intake Estimated with DH-E versus 24-hour Recalls

The Pearson coefficients between the DH-E2 and the mean of the seven 24-hour recalls were higher than 0.35 in all food groups (Table 3). The correlation coefficients of the principal food groups were: cereals (r = 0.66), meat (r = 0.66), fish (r = 0.42), vegetables (r = 0.62) and fruits (r = 0.44). The mean coefficient across all groups was 0.53. The correlation coefficients for the main macronutrients were: energy (r = 0.76), proteins (r = 0.58), lipids (r = 0.73), saturated fatty acids (SFA) (r = 0.73), monounsaturated fatty acids (MUFA) (r = 0.59), polyunsaturated fatty acids (PUFA) (r = 0.57) and carbohydrates (r = 0.66). Vitamin A (r = 0.26) and vitamin D (r = 0.30) had the lowest correlation coefficients. The mean correlation coefficient for nutrients and vitamins was 0.55 (Table 3). Similar results were obtained after adjusting for energy, and when calculating intra-class correlation coefficients and de-attenuation coefficients (data not shown in the latter case).

**Table 3:** Validity of food and nutrient intake estimated by DH-E2 (at 12 months from baseline) versus mean of seven 24-hour recalls during one year.

	Pearson Correlation coefficient		Intraclass correlation coefficient	
	Unadjusted	Energy-adjusted	Unadjusted	Energy-adjusted
**Food groups**				
Cereals	0.66	0.63	0.65	0.62
Milk	0.68	0.69	0.67	0.69
Meat	0.66	0.61	0.66	0.61
Eggs	0.49	0.49	0.41	0.41
Fish	0.42	0.42	0.35	0.36
Oils and fats	0.47	0.46	0.47	0.46
Vegetables	0.62	0.60	0.45	0.52
Legumes	0.35	0.35	0.22	0.26
Tubers	0.36	0.36	0.35	0.34
Fruits	0.44	0.42	0.42	0.41
Dried fruits and nuts	0.43	0.43	0.43	0.43
Chocolate and similar	0.49	0.49	0.47	0.49
Coffee, cocoa and infusions	0.73	0.71	0.71	0.70
Soft drinks	0.42	0.40	0.42	0.40
Alcoholic beverages	0.65	0.64	0.62	0.63
**Nutrients**				
Energy	0.76	–	0.75	–
Total protein	0.58	0.50	0.58	0.49
Animal protein	0.62	0.59	0.62	0.59
Vegetable protein	0.62	0.60	0.59	0.59
Fats	0.73	0.65	0.73	0.64
Saturated fatty acids	0.73	0.63	0.73	0.63
Monounsaturated fatty acids	0.59	0.51	0.60	0.51
Polyunsaturated fatty acids	0.57	0.43	0.58	0.43
Linoleic acid (C 18∶2, n-6), (g/d)	0.59	0.44	0.59	0.45
α-linolenic acid (C 18∶3, n-3), (g/d)	0.49	0.47	0.45	0.45
Eicosapentanoic acid, EPA (C 20∶5, n-3), (g/d)	0.55	0.56	0.53	0.55
Docosapentanoic acid, DPA (C 22∶5, n-3), (g/d)	0.54	0.52	0.51	0.51
Docosahexanoic acid, DHA (C 22∶6, n-3), (g/d)	0.60	0.60	0.54	0.57
*Trans* FA	0.56	0.48	0.55	0.47
Cholesterol	0.64	0.57	0.64	0.56
Total carbohydrates	0.66	0.61	0.65	0.61
Sugars	0.55	0.52	0.55	0.52
Polysaccharides	0.68	0.65	0.67	0.65
Ethanol	0.69	0.69	0.63	0.66
Fiber	0.49	0.52	0.44	0.51
Caffeine	0.47	0.47	0.40	0.42
Sodium	0.56	0.47	0.56	0.47
Potassium	0.43	0.43	0.43	0.43
Calcium	0.50	0.49	0.48	0.48
Magnesium	0.46	0.46	0.46	0.46
Phosphorus	0.62	0.58	0.62	0.58
Iron	0.49	0.46	0.48	0.45
Zinc	0.55	0.45	0.55	0.45
Selenium	0.42	0.41	0.40	0.40
Iodine	0.47	0.45	0.45	0.45
Vitamin A	0.26	0.27	0.24	0.25
Retinoids	0.50	0.43	0.47	0.40
Carotenoids	0.43	0.45	0.39	0.44
Vitamin D	0.30	0.29	0.30	0.29
Vitamin E	0.52	0.47	0.52	0.47
Thiamin	0.54	0.44	0.49	0.43
Riboflavin	0.60	0.57	0.58	0.56
Niacin	0.55	0.46	0.54	0.46
Vitamin B6	0.50	0.49	0.50	0.50
Folic acid	0.46	0.48	0.46	0.48
Vitamin B12	0.47	0.44	0.44	0.42
Vitamin C	0.66	0.65	0.66	0.65

There was little gross misclassification between the DH-E and the 24-hour recalls (Table 4). The mean percentage of subjects simultaneously classified in the lowest quintile of the 24-h recalls and in the highest quintile of the DH-E was 3.7%, while it was 5.4% for the opposite situation. In all food and nutrient groups, a mean of 71.4% of subjects were classified in the DH-E within one quintile of the 24-h recalls.

**Table 4:** Cross-classification of subjects by food and nutrient intake estimated with DH-E2 (at 12 months from baseline) and mean of seven 24-hour recalls during one year.

	Lowest quintile in 7 dietary recalls and highest quintile inDH-E2	Highest quintile in 7 dietary recalls and lowest quintile inDH-E2	Classified in DH2 within one quintile in 7 dietary recalls
**Food groups**			
Cereals	0.0	0.0	70.4
Milk	5.3	0.0	83.7
Meat	0.0	0.0	69.4
Eggs	5.3	10.5	63.3
Fish	0.0	10.5	77.6
Oils and fats	0.0	5.3	61.2
Vegetables	10.5	0.0	72.4
Legumes	0.0	10.5	68.4
Tubers	21.1	5.3	65.3
Fruits	0.0	10.5	79.6
Dried fruits and nuts	8.7	0.0	72.7
Chocolate and similar	0.0	5.3	72.2
Coffee, cocoa and infusions	6.0	15.0	77.9
Soft drinks	8.0	10.5	71.9
Alcoholic beverages	3.5	0.0	77.3
**Nutrient**			
Energy	0.0	0.0	78.8
Total protein	0.0	0.0	67.3
Animal protein	5.0	0.0	68.3
Vegetable protein	5.0	5.0	72.1
Fats	5.0	5.0	76.0
Saturated fatty acids	5.0	0.0	82.7
Monounsaturated fatty acids	0.0	10.0	71.2
Polyunsaturated fatty acids	5.0	0.0	66.3
Linoleic acid (C 18∶2, n-6)	5.0	0.0	66.3
α-linolenic acid (C 18∶3, n-3)	0.0	0.0	71.2
Eicosapentanoic acid, EPA (C 20∶5, n-3)	0.0	10.0	70.2
Docosapentanoic acid, DPA (C 22∶5, n-3)	5.0	5.0	74.0
Docosahexanoic acid, DHA (C 22∶6, n-3)	0.0	5.3	66.3
*Trans* FA	0.0	5.0	73.1
Cholesterol	5.0	0.0	71.2
Total carbohydrates	5.0	0.0	72.1
Sugars	0.0	0.0	76.9
Polysaccharides	0.0	0.0	69.2
Ethanol	0.0	0.0	81.7
Fiber	5.0	5.0	74.0
Caffeine	0.0	5.0	74.0
Sodium	0.0	5.3	66.3
Potassium	5.3	10.0	63.5
Calcium	5.0	0.0	63.5
Magnesium	5.3	15.0	72.1
Phosphorus	0.0	0.0	69.2
Iron	5.0	15.0	63.5
Zinc	0.0	10.0	65.4
Selenium	0.0	15.0	67.3
Iodine	10.5	15.0	73.1
Vitamin A	15.8	10.0	66.3
Retinoids	5.0	0.0	76.0
Carotenoids	5.0	10.0	74.0
Vitamin D	5.0	5.3	62.5
Vitamin E	5.0	5.0	70.2
Thiamine	15.8	5.0	67.3
Riboflavin	5.3	5.0	75.0
Niacin	0.0	15.0	74.0
Vitamin B6	5.0	15.0	76.9
Folic acid	0.0	15.0	72.1
Vitamin B12	0.0	0.0	73.1
Vitamin C	0.0	0.0	73.1

### Validity of Nutrient Intake Estimated with DH-E versus Biomarkers

The correlation coefficients between the DH-E2 and the biomarkers were low SFA (r = 0.03), and MUFA (r = 0.08), but were higher for eicosapentanoic acid (EPA, 20∶5*n*-3, r = 0.46), docosahexanoic acid (DHA, 22∶6*n*-3, r = 0.36), EPA+DHA (also known as the omega-3 index, r = 0.40), and urea nitrogen versus total protein (r = 0.36) (Table 5).

**Table 5:** Validity of intake of some nutrients with DH-E2 (at 12 months from baseline) versus biomarkers (mean of measurements at 0, 6 and 12 months).

Biomarkers	Pearson Correlation coefficient
**Total fatty acids in blood**	
Saturated fatty acids	0.03
Monounsaturated fatty acids	0.08
Omega-3 Index	0.40
Linoleic acid (C 18∶2, n-6)	0.15
α-linolenic acid (C 18∶3, n-3)	0.15
Eicosapentanoic acid, EPA (C 20∶5, n-3)	0.46
Docosapentanoic acid, DPA (C 22∶5, n-3)	0.13
Docosahexanoic acid, DHA (C 22∶6, n-3)	0.36
**Serum vitamins**	
Vitamin A	0.12
Vitamin C	0.28
Vitamin E/cholesterol	0.13
**Other serum tests**	
Calcium	0.18
**24 hour urine tests**	
Sodium	0.19
Potassium	0.17
Urea nitrogen vs. total protein	0.36

### Reproducibility of Food and Nutrient Intake Estimated with DH-E

The ICC for the most important food groups were: cereals (ICC = 0.59), meat (ICC = 0.58), fish (ICC = 0.38), vegetables (ICC = 0.55) and fruits (ICC = 0.40). The mean coefficient for all food groups was 0.45. The correlation coefficients for the main macronutrients were: energy (ICC = 0.66), proteins (ICC = 0.32), lipids (ICC = 0.57), SFA (ICC = 0.65), MUFA (ICC = 0.50), PUFA (ICC = 0.37) and carbohydrates (ICC = 0.58). Iron and vitamin B12 had the lowest coefficients (ICC = 0.14 and ICC = 0.15, respectively). The mean ICC for all nutrients was 0.45 (Table 6).

**Table 6:** Reproducibility of DH-E. Correlation coefficients between DH-E1 (at study baseline) and DH-E2 (at 12 months from baseline).

	Pearson Correlation coefficient		Intraclass correlation coefficient	
	Unadjusted	Energy-adjusted	Unadjusted	Energy-adjusted
**Food groups**				
Cereals	0.61	0.56	0.59	0.54
Milk	0.60	0.59	0.58	0.59
Meat	0.61	0.59	0.58	0.55
Eggs	0.42	0.41	0.34	0.35
Fish	0.40	0.41	0.38	0.39
Oils and fats	0.30	0.31	0.26	0.30
Vegetables	0.59	0.59	0.55	0.55
Legumes	0.42	0.42	0.37	0.38
Tubers	0.08	0.04	0.08	0.04
Fruits	0.51	0.49	0.40	0.40
Dried fruits and nuts	0.40	0.40	0.35	0.39
Chocolate and similar	0.41	0.40	0.42	0.41
Coffee, cocoa and infusions	0.78	0.77	0.77	0.75
Soft drinks	0.45	0.44	0.44	0.43
Alcoholic beverages	0.60	0.59	0.60	0.59
**Nutrients**				
Energy	0.69	–	0.66	–
Total protein	0.43	0.33	0.32	0.22
Animal protein	0.63	0.59	0.61	0.57
Vegetable protein	0.50	0.45	0.44	0.43
Lipids	0.58	0.51	0.57	0.49
Saturated fatty acids	0.65	0.57	0.65	0.56
Monounsaturated fatty acids	0.52	0.46	0.50	0.44
Polyunsaturated fatty acids	0.39	0.25	0.37	0.24
Linoleic acid (C 18∶2, n-6)	0.40	0.26	0.38	0.25
α-linolenic acid (C 18∶3, n-3)	0.27	0.26	0.28	0.26
Eicosapentanoic acid, EPA (C 20∶5, n-3)	0.40	0.40	0.40	0.40
Docosapentanoic acid, DPA (C 22∶5, n-3)	0.50	0.48	0.51	0.49
Docosahexanoic acid, DHA (C 22∶6, n-3)	0.40	0.40	0.40	0.40
*Tran* FA	0.53	0.42	0.51	0.41
Cholesterol	0.54	0.48	0.52	0.46
Total carbohydrates	0.66	0.61	0.58	0.57
Sugars	0.50	0.47	0.45	0.46
Polysaccharides	0.62	0.58	0.60	0.57
Ethanol	0.63	0.62	0.62	0.61
Fiber	0.49	0.51	0.47	0.50
Caffeine	0.40	0.41	0.40	0.41
Sodium	0.45	0.34	0.45	0.34
Potassium	0.52	0.54	0.47	0.52
Calcium	0.48	0.43	0.47	0.42
Magnesium	0.53	0.50	0.44	0.46
Phosphorus	0.53	0.45	0.50	0.43
Iron	0.28	0.19	0.14	0.10
Zinc	0.60	0.48	0.57	0.47
Selenium	0.35	0.31	0.31	0.30
Iodine	0.37	0.34	0.37	0.34
Vitamin A	0.20	0.19	0.19	0.19
Retinoids	0.45	0.35	0.44	0.35
Carotenoids	0.46	0.46	0.45	0.45
Vitamin D	0.21	0.19	0.17	0.17
Vitamin E	0.47	0.45	0.44	0.43
Thiamin	0.45	0.34	0.38	0.30
Riboflavin	0.63	0.56	0.59	0.55
Niacin	0.53	0.47	0.49	0.43
Vitamin B6	0.42	0.35	0.32	0.29
Folic acid	0.32	0.33	0.29	0.31
Vitamin B12	0.17	0.12	0.15	0.12
Vitamin C	0.52	0.51	0.51	0.50
Caffeine	0.40	0.41	0.40	0.41

## Discussion

The DH-E has shown good validity and reproducibility in estimating food and nutrient intake. Specifically, the validity of the DH-E was similar to that of other instruments used to measure the usual diet in Spain [3], [10]–[12], [32]–[34], and of other DH outside of Spain [6], [35]–[38].

The correlations for nutrient intake between the DH-E and the 24-hour recalls were generally higher than 0.40, which indicates moderate agreement and permits reliable classification of subjects [39]. The correlation coefficients for calcium, iron, zinc, selenium and iodine were similar to those obtained in a systematic review in which the FFQ was the most frequently used method of dietary assessment [40]. Both validity and reproducibility are expected to be lower for nutrients than for foods, because some nutrients like vitamin A and D are found in only a few foods and in a relatively high concentration, and because for some nutrients such as vitamin A, iron and linoleic acid, intra-subject variation is usually higher than inter-subject variation [41]. On the other hand, our results were stable because the correlation coefficients were unchanged after adjusting for energy [3] or de-attenuation [34].

When total blood FA were used in the validation, our results were similar to those of other studies [42], [43], as expected, the results were better for essential nutrients like some polyunsaturated FA, and worse for non-essential FA like saturated and monounsaturated FA. The results for eicosapentanoic acid were comparable to those of studies that measured it in fat aspirates [44]. Our results in the case of vitamin C were also similar to those of previous studies, but were somewhat lower for vitamins A and E [45]. Finally, in comparison with other validation studies [46] our results were similar for urinary sodium, urinary potassium and urea nitrogen.

The reproducibility of the DH-E was similar to that obtained with other DHs [10]–[12], [38] or that obtained with FFQs [47], [48]. In general, better results were obtained when the DH-E2 was correlated with the mean of the seven 24-h recalls than with the DH-E1. Given that reproducibility depends on both true intake variation and measurement errors, we cannot rule out the possibility that some variation is due to the fact that the dietary information referred to different periods, or to a “learning effect” resulting from the experience acquired by the interviewer and respondents during the year. On the other hand, in comparison with instruments like the DH, which are flexible with regard to the number of foods considered or quantification of portion size, reproducibility is usually higher in instruments like FFQs, which limit the variety of foods, and in those that estimate portion size based on a single standard reference portion.

This study has some strengths. Specifically, it meets the quality criteria recommended in validation studies [49]:sufficient sample size, heterogeneous sample, data collection by interviewers, and consideration of seasonality. Moreover, we had information on a large number of foods and nutrients, including foods consumed locally and those consumed exclusively in Spain. In contrast with the frequent criticism that the DH may not be standardized [50],data collection with the DH-E follows a systematic process in which the interviewers have been trained. It should also be noted that the DH-E was structured by mealtimes, which makes it easier to obtain information and produces better results than when structured by food groups [51]. An additional advantage of the DH is that it prevents interviewers from forgetting specific foods and helps to include day-by-day variability in food consumption.

Among the study limitations is an insufficient number of 24-h recalls and biological samples to validate some nutrients with high intra-subject variability (like vitamins A or D), and the lack of biomarkers of long-term consumption. Moreover, we cannot rule out some “learning effect” in data collection, and a “recency effect” [52] in reporting information, since recall of past food consumption may be distorted by current consumption.

In conclusion, the DH-E has good validity and reproducibility in the estimation of food and nutrient intake and may be useful to collect food information in epidemiological studies in Spain.
